# Osmotic pressure‐induced calcium response states

**DOI:** 10.1002/2211-5463.70094

**Published:** 2025-08-29

**Authors:** Zidan Gao, Keiji Naruse, Masatoshi Morimatsu

**Affiliations:** ^1^ Department of Cardiovascular Physiology, Okayama University Graduate School of Medicine, Dentistry and Pharmaceutical Sciences Okayama Japan; ^2^ Faculty of Medicine, Dentistry and Pharmaceutical Sciences Okayama University Okayama Japan

**Keywords:** calcium wave, Connexin 43, hypotonic pressure, osmotic pressure, ryanodine receptor

## Abstract

Osmotic pressure is essential for maintaining cellular homeostasis; however, the mechanisms by which cells sense and respond to acute osmotic stress remain incompletely understood. Here, we applied rapid osmotic pressure stimulation to cultured HEK293T cells and observed dynamic intracellular calcium responses. Acute hypotonic stimulation evoked calcium response patterns, whereas hypertonic and isotonic stress did not elicit similar effects. Mechanistically, these calcium signals originated from the endoplasmic reticulum via ryanodine receptor 2 and propagated to neighboring cells through Connexin 43‐mediated gap junctions. These findings reveal a previously unrecognized role for calcium signaling in the acute cellular response to osmotic stress, providing new insights into the mechanisms of intercellular communication during osmotic adaptation.

## Graphical abstract

Acute hypotonic stress triggers distinct calcium response patterns in HEK293T cells. These signals originate from the endoplasmic reticulum via RyR2 and propagate to neighboring cells through gap junctions. This result highlights a novel calcium‐dependent mechanism underlying the intracellular and intercellular response to osmotic pressure.

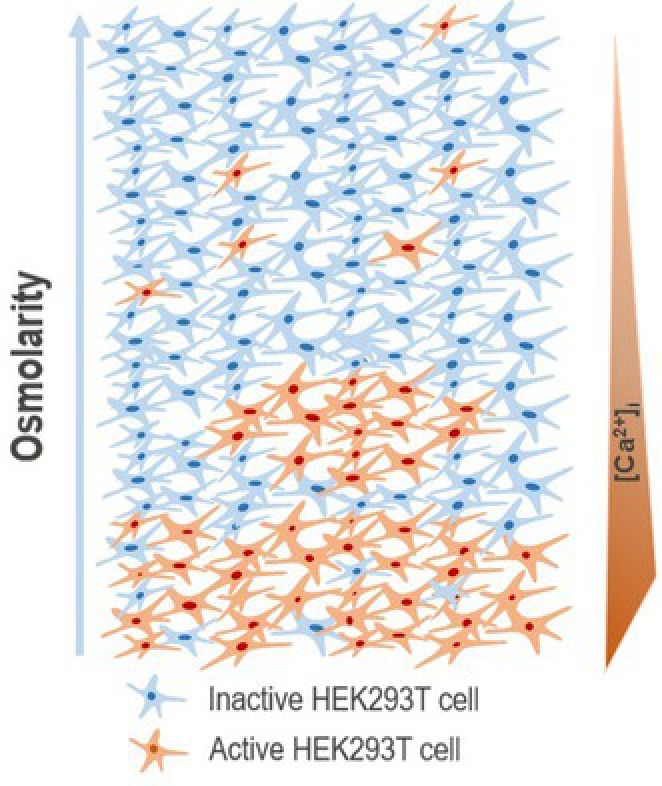

**Abbreviations**
ANT: adenine nucleotide translocase
AQP3: aquaporin 3
AQP4: aquaporin 4
[Ca^2+^]i: intracellular calcium concentration
Ca^2+^
: calcium ion
Cx43: connexin 43
EGTA: ethylene glycol‐bis(β‐aminoethyl ether)‐N,N,N′,N′‐tetraacetic acid
ER: endoplasmic reticulum
FCCP: carbonyl cyanide‐p‐trifluoromethoxyphenylhydrazone
HEK: human embryonic kidney cells
HUVEC: human umbilical vein endothelial cells
IP_3_
: Inositol 1,4,5‐trisphosphate
KD: knockdown
KO: knockout
MDCK: Madin–Darby canine kidney cells
MLC: myosin light chain
MTPT: mitochondrial permeability transition pore
NA: not applicable
OSCARS: osmotic pressure‐induced calcium response states
PI3K/AKT: phosphoinositide 3‐kinase/protein kinase B pathway
PLC: phospholipase C
RPTEC: renal proximal tubule epithelial cells
RyR2: ryanodine receptor 2
SACs: selective inhibitor of stretch‐activated ion channels
SIAD: syndrome of inappropriate antidiuresis
VGCC: voltage‐gated calcium channel

Cells detect and respond to mechanical environmental stimuli, such as stiffness, shear stress, and stretch [1]. Osmotic pressure is one of these mechanical stimuli and an essential physiological process for maintaining homeostasis [2]. Osmotic dysregulation can also lead to certain diseases. For example, hyperglycemic hyperosmolar state and diabetes insipidus are kidney diseases associated with hypertonicity. The most common hypotonic osmolar disorder is a syndrome of inappropriate antidiuresis (SIAD) [3, 4]. The physiological osmolarity of human plasma extracellular fluid is 275–295 mOsm·kg^−1^ H_2_O [5]. However, in pathological states, such as inflammation, ischemia, or tumor microenvironments, extracellular osmotic pressure may significantly increase due to the accumulation of solutes, cytokines, lactate, and cell debris [6, 7, 8]. For instance, studies have reported that tumor interstitial osmolarity may exceed 330–350 mOsm·kg^−1^ H_2_O, and renal medullary interstitial fluid can reach up to 1200 mOsm·kg^−1^ H_2_O during countercurrent concentration [9]. Furthermore, acute osmotic changes regulate cellular functions, such as cell proliferation, apoptosis, metabolism, epithelial transport, and migration [10]. A hypotonic solution induces an influx of water molecules into the extracellular solution [11]. This process results in cell volume expansion. In contrast, a hypertonic solution shrinks the cellular volume due to the outflow of water molecules into the extracellular solution [12]. Under isotonic conditions, cell volume remains stable. Drastic changes in the cellular environment trigger functional adaptations; however, the mechanisms by which cells sense and transduce signals in response to acute osmotic pressure changes remain unclear. Calcium ions (Ca^2+^) are critical mediators in several signaling pathways [13, 14, 15]. Some groups have reported that hypotonic conditions induce the fluctuation (oscillation or shock) of intracellular calcium concentration ([Ca^2+^]i) in chondrocytes, endothelial cells, glioma cells, and odontoblasts [16, 17, 18, 19]. Despite its relevance, the calcium signaling responses to graded osmotic stress are also poorly defined. This study investigated the cellular response to acute osmotic changes and reported the osmotic calcium response states.

## Materials and methods

### Cell culture

HEK293T cells and Piezo KO HEK293T cells were gifted by Dr. Nonomura (Kyoto University). Human umbilical vein endothelial cells (HUVEC; Lonza, Basel, Switzerland), Madin–Darby canine kidney cells (MDCK/NBL‐2; Japanese Collection of Research Bioresources [JCRB], Osaka, Japan), and human renal proximal tubule epithelial cells (RPTEC/TERT1; ATCC, Manassas, VA, USA) were cultured following the manufacturer's instructions. DMEM medium (No. 043–30085; Fujifilm Wako, Osaka, Japan) with 10% fetal bovine serum (FBS) (Sigma‐Aldrich, St.Louis, MO, USA) and 0.02% penicillin–streptomycin solution (Sigma‐Aldrich, St. Louis, MO, USA) was used for normal cell culture. This standard cell medium is ‘an isotonic solution’ in this study. Cells were cultured in a 100 mm plastic plate (TPP, Trasadingen, Swizerland) at 37 °C and 5% CO_2_ in an incubator. HEK 293 T cells were subcultured twice a week at 0.2 and 0.3 × 10^6^ cells·mL^−1^ densities. After coating the surface of the 48‐well plate with 0.1% fibronectin (CORNING, Corning, NY, USA), cells were seeded in the 48‐well plate (TPP, Trasadingen, Swtizerland) at a density of 1 × 10^5^ cells·mL^−1^ and cultured overnight before the osmolarity experiment.

### Preparation of hypertonic and hypotonic solutions

100 mm sucrose (Fujifilm Wako, Osaka, Japan) was added to DMEM with FBS to make a hypertonic solution. In the case of a hypotonic solution, we mixed the DMEM with distilled water at several concentrations and maintained CaCl_2_ and Mg_2_SO_4_ at 200 mg·L^−1^ and 97.67 mg·L^−1^, respectively (followed by the original DMEM composition). For example, a 50 % hypotonic solution consisted of 50 % distilled water, 30 % DMEM, 10 % FBS, 2 % HEPES (pH 7.4, NaOH), CaCl_2_ and Mg_2_SO_4_. Calcium (−) DMEM (Nacalai Tesque, Kyoto, Japen) was used for calcium‐free conditions. An automatic freezing point osmometer (OM815; VOGEL, Fenwald, Germany) was used to measure the osmolarity of all solutions.

### Imaging of intracellular calcium concentration in HEK 293 T cells

For intracellular [Ca^2+^]i imaging, the cells were incubated with 4.5 μm Cal‐520 dye (ATT Bioquest, Sunnyvale, CA, USA) for 30 min, and, after removing the medium, a hypotonic/hypertonic solution was slowly added along the bottom wall. Microscopic observations were made with a microscope (IX‐83; Evident, Tokyo, Japan). Bright‐field and epifluorescence images were recorded with a sCMOS camera (ORCA‐Fusion BT; Hamamatsu, Hamamatsu, Japan). All microscopic images were analyzed using the fiji/imagej software (https://imagej.net/Fiji).

### Blocking the function of proteins with chemical inhibitors and siRNA knockdown

Cells were incubated with chemical inhibitors before acute osmotic pressure stimulation (see details in Table 2). HEK293T cells were transfected at 70 % confluence using Lipofectamine RNAiMAX Transfection Reagent (Thermo Fisher Scientific, Waltham, MA, USA). Ryanodine receptor 2 (RyR2) siRNA duplex (NIPPON GENE, Tokyo, Japan) was added according to the manufacturer's protocol.

### RNA extraction, cDNA synthesis, and RT‐PCR analysis

Total RNA was extracted and purified using the Monarch Total RNA Miniprep Kit (New England Biolabs, Ipswich, MA, USA) per the manufacturer's instructions. cDNA was then synthesized using the ReverTra Ace qPCR RT Master Mix with gDNA Remover (TOYOBO, Osaka, Japan) according to the manufacturer's instructions. The gene expression level was analyzed using real‐time PCR, which was performed with KAPA SYBR FAST dye (Kapa Biosystems, Wilmington, MA, USA) under the 96‐well cycler qTOWER^3^G Real‐Time PCR System (Analytik Jena, Jena, Germany).

RyR2 Primer sequence (5′ → 3′):

Forward primer (CTTGAGGTTGGCTTTCTGCCAG), Reverse primer(TGTGCCAGCAAAGAGAGGAGCA).

GAPDH Primer sequence (5′ → 3′):

Forward primer (GTCTCCTCTGACTTCAACAGCG), Reverse primer (ACCACCCTGTTGCTGTAGCCAA).

### Fluorescence images of RyR2 and endoplasmic reticulum localization in fixed HEK293T cells

Cells were cultured on a glass‐bottom dish (AGC Thechno Glass, Shizuoka, Japan) and fixed with 4% paraformaldehyde. RyR2 was stained with an anti‐RyR2 antibody (Proteintech, Rosemont, IL, USA, 1 : 400) and secondary antibody (Invitrogen, Carlsbad, CA, USA, 1 : 1000). Endoplasmic reticulum (ER) was stained with ER indicator (ER Seeing;Funakoshi, Tokyo, Japan). After stimulation with a 50 % hypotonic solution, cells were incubated for 5 min in the incubator and then fixed with 4 % paraformaldehyde. Microscopic observations were performed with a confocal microscope (FV3000; Evident, Tokyo, Japan). Images were deconvolved using cellSens (Evident, Tokyo, Japan).

### Velocity of calcium wave

For each sample, one wave (usually the one closest to the center) was tracked, and the difference between the wave's initial fluorescence intensity area and its final fluorescence intensity area was taken as an apparent circular shape to calculate the radius using the circle calculation formula. Finally, velocity was calculated using the following formula:

$$\text{Velocity}=\sqrt{\left(\frac{\text{Area}2-\text{Area}1}{\pi }\right)}/t$$

### Statistical analysis

For comparisons between two groups, an unpaired two‐tailed Student's *t*‐test was used for normally distributed data, while the Mann–Whitney *U*‐test was applied for non‐normally distributed data. For multiple group comparisons, one‐way ANOVA followed by Tukey's *post hoc* test was used for normally distributed data, and the Kruskal–Wallis test followed by Dunn's *post hoc* test was used for nonparametric data. Statistical significance was set at *P* ≤ 0.05 (GraphPad Prism 5). Supplement part figures applied in R (v4.5.0) using the ggplot2 package.

## Results

### Osmotic pressure induces morphological change and regulates intracellular calcium concentration

**Fig. 1:**
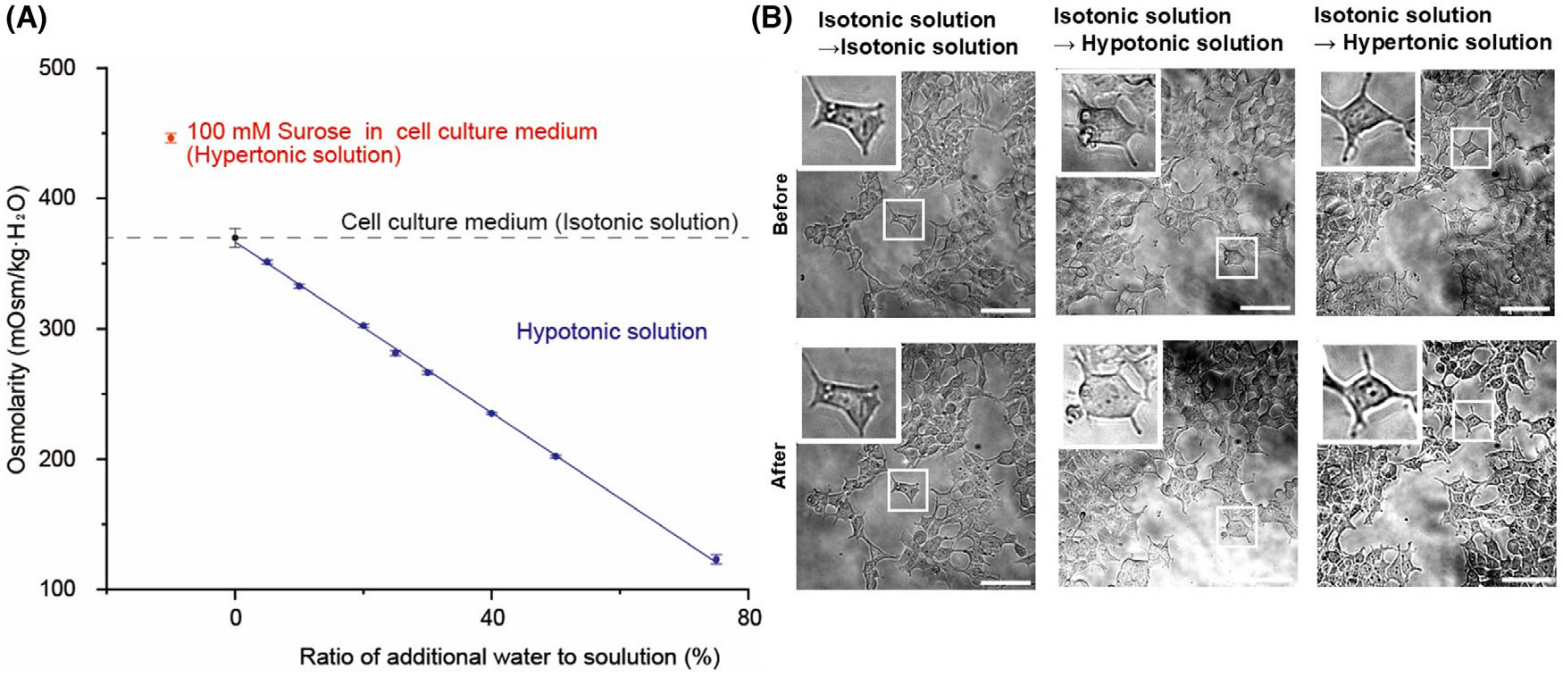
Osmolarity in each solution and morphological response of HEK293T cells to different osmotic conditions. (A) The osmolarity of each solution was measured by osmometer (*n* = 3 trials in each condition). Error bars represent standard deviation. (B) The cell morphologies of HEK293T cells during the change of the cells' standard medium to isotonic solution (~370 mOsm·kg^−1^·H_2_O), hypotonic solution (~200 mOsm·kg^−1^·H_2_O), and hypertonic solution (~450 mOsm·kg^−1^·H_2_O) were observed under the microscope. There was no change in cellular morphology before and after exposure to the isotonic solution. Hypotonic pressure caused the cells to expand, while hypertonic pressure caused them to shrink within approximately 5 s. The scale bar is 20 μm.

We prepared several osmotic solutions to stimulate HEK293T cells and measured the osmolarity values across several experimental conditions (Table 1, Fig. 1A, Methods). The osmolarity of our standard cell culture medium with FBS was approximately 370 mOsm·kg^−1^ H_2_O, which is higher than the osmolarity of human plasma (285 mOsm·kg^−1^ H_2_O) (Table 1). The addition of the isotonic solution did not cause any changes to the cell morphology. However, cells expanded in low osmotic pressure conditions and shrank in high osmotic pressure conditions (Fig. 1B, Fig. S1).

**Table 1:** Standard osmotic pressure solutions.

	Osmolarity (mOsm·kg^−1^·H_2_O)	Composition					
Hypertonic solutions	446.3	Addition of 100 mm sucrose in normal cell medium					
Isotonic solutions	369.7	Normal cell culture medium					
		Distilled water (%)	D‐MEM (%)	Hepes Buffer‐NaOH 7.4 (%)	FBS (%)	CaCl_2_ (%)	MgSO_4_ (%)
Hypotonic solutions 330 mOsm·kg^−1^·H_2_O	332.7	10	70	2	10	4*	4**
Hypotonic solutions 300 mOsm·kg^−1^·H_2_O	302.3	20	60	2	10	4*	4**
Hypotonic solutions 270 mOsm·kg^−1^·H_2_O	266.3	30	50	2	10	4*	4**
Hypotonic solutions 235 mOsm·kg^−1^·H_2_O	235	40	40	2	10	4*	4**
Hypotonic solutions 200 mOsm·kg^−1^·H_2_O	202	50	30	2	10	4*	4**
Hypotonic solutions 120 mOsm·kg^−1^·H_2_O	123	75	5	2	10	4*	4**

Hypotonic solutions are shown with different formulas, and adjusted CaCl_2_ and MgSO_4_ account for 200 mg·L^−1^ and 97.67 mg·L^−1^ (each account for 4% of the total). The osmolarity values presented in this article have been rounded to the nearest 10 (e.g., hypertonic solution: ~450 mOsm·kg^−1^·H_2_O; isotonic Solution: ~370 mOsm·kg^−1^·H_2_O).

* Final concentration is 200 mg·L^−1^

** Final concentration is 97.67 mg·L^−1^.

**Fig. 2:**
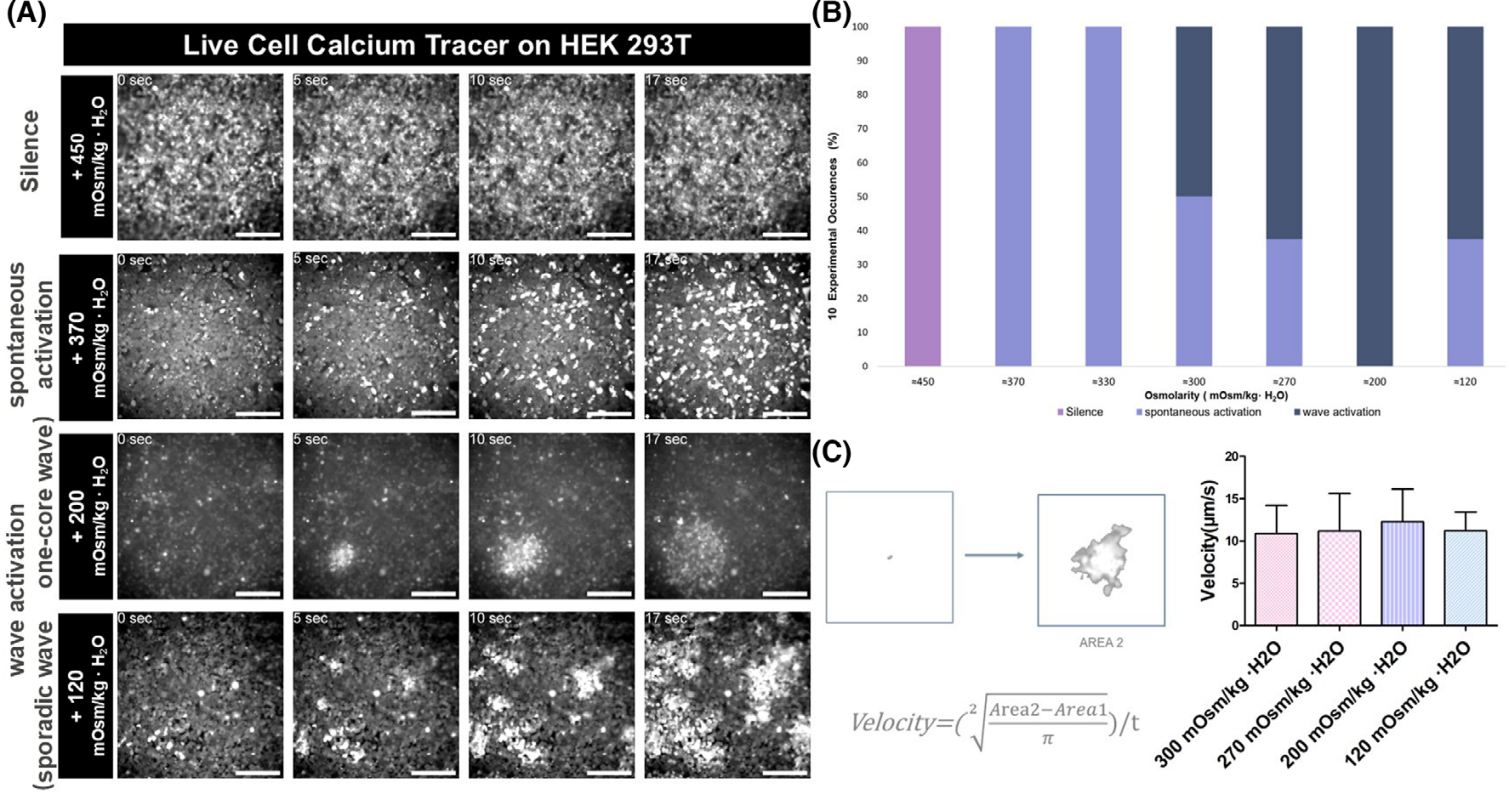
Osmotic pressure‐induced calcium response (OSCARS) states. (A) Imaging of intracellular calcium concentration. OSCARS: Silence mode—response to hypertonic solution (~450 mOsm·kg^−1^·H_2_O): No calcium activation was observed after the stimulation with a hypertonic solution. Spontaneous activation mode—response to isotonic solution (~370 mOsm·kg^−1^·H_2_O): Cells exhibited an auto‐activation response upon adding an isotonic solution, displaying a sparking‐like activation pattern. Wave activation mode‐hypotonic solution (< 300 mOsm·kg^−1^·H_2_O): Cell activation started in one cell and spread to neighboring cells, showing activation spreading as a wave. The scale bar is 200 μm. (B) Bar chart indicates the rate of OSCARS (10 trials in each experiment). The 450 mOsm·kg^−1^·H_2_O solution showed only the silent mode response, and the 200 mOsm·kg^−1^·H_2_O solution showed only the wave activation. The spontaneous mode was observed in all osmotic solutions except the two above. (C) The shape of the initial and final calcium waves was analyzed, and the velocity was evaluated by considering the duration of the calcium waves using the formula. The velocity of 4 different hypotonic solutions was measured, and the average velocity of each group was calculated (one‐way ANOVA, mean ± SEM, *n* = 5). The results were not statistically significant.

**Fig. 3:**
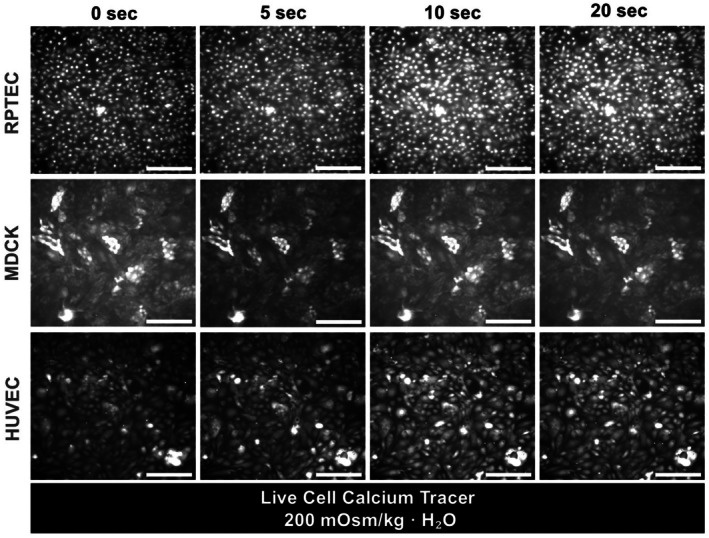
Hypotonic stimulation induces only spontaneous calcium activation in renal proximal tubule epithelial cells (RPTEC), Madin–Darby canine kidney cells (MDCK), and human umbilical vein endothelial cells (HUVEC). Approximately 200 mOsm·kg^−1^·H_2_O solutions stimulate RPTEC, MDCK cells, and HUVEC. We observed only calcium spontaneous activation mode in these cells. The scale bar is 200 μm.

Next, we observed the cellular calcium response to acute osmotic pressure changes (Fig. 2A). Hypertonic conditions (~450 mOsm·kg^−1^ H_2_O) did not increase [Ca^2+^]i (silent mode) (Fig. 2A, Fig. S2). Under isotonic conditions (~370 mOsm·kg^−1^ H_2_O), cells displayed a spontaneous calcium activation reaction (spontaneous activation mode) (Fig. 2A, Fig. S2). Under hypotonic conditions (< 300 mOsm·kg^−1^ H_2_O), cells displayed a wave‐like calcium activation for over a few minutes. Some showed a rapid, sporadic wave activation mode, while others displayed a one‐core wave activation mode (we defined these as wave activation modes) (Fig. 2A, Video S1, Fig. S2). We named these three modes osmotic pressure‐induced calcium response states (OSCARS). The OSCARS stimulated by solutions with different osmotic pressures were counted (Fig. 2B). As the osmotic pressure declines, the frequency of the spontaneous activation mode concomitantly decreases, while the frequency of the wave activation mode increases until approximately 200 mOsm·kg^−1^ H_2_O. The velocity of calcium wave activation mode is 12.3 ± 3.9 μm·s^−1^ under the ~200 mOsm·kg^−1^ H_2_O condition (Fig. 2C, Methods). In addition, the velocity of calcium waves exhibited no statistically significant variations across a range of osmotic pressure conditions (Fig. 2C). Moreover, we found that MDCK cells, RPTECs, and HUVECs did not show the calcium wave activation modes under the same hypotonic condition as the HEK293T experiments (Fig. 3, Fig. S3).

### Low osmotic pressure induces calcium wave activation mode

**Fig. 4:**
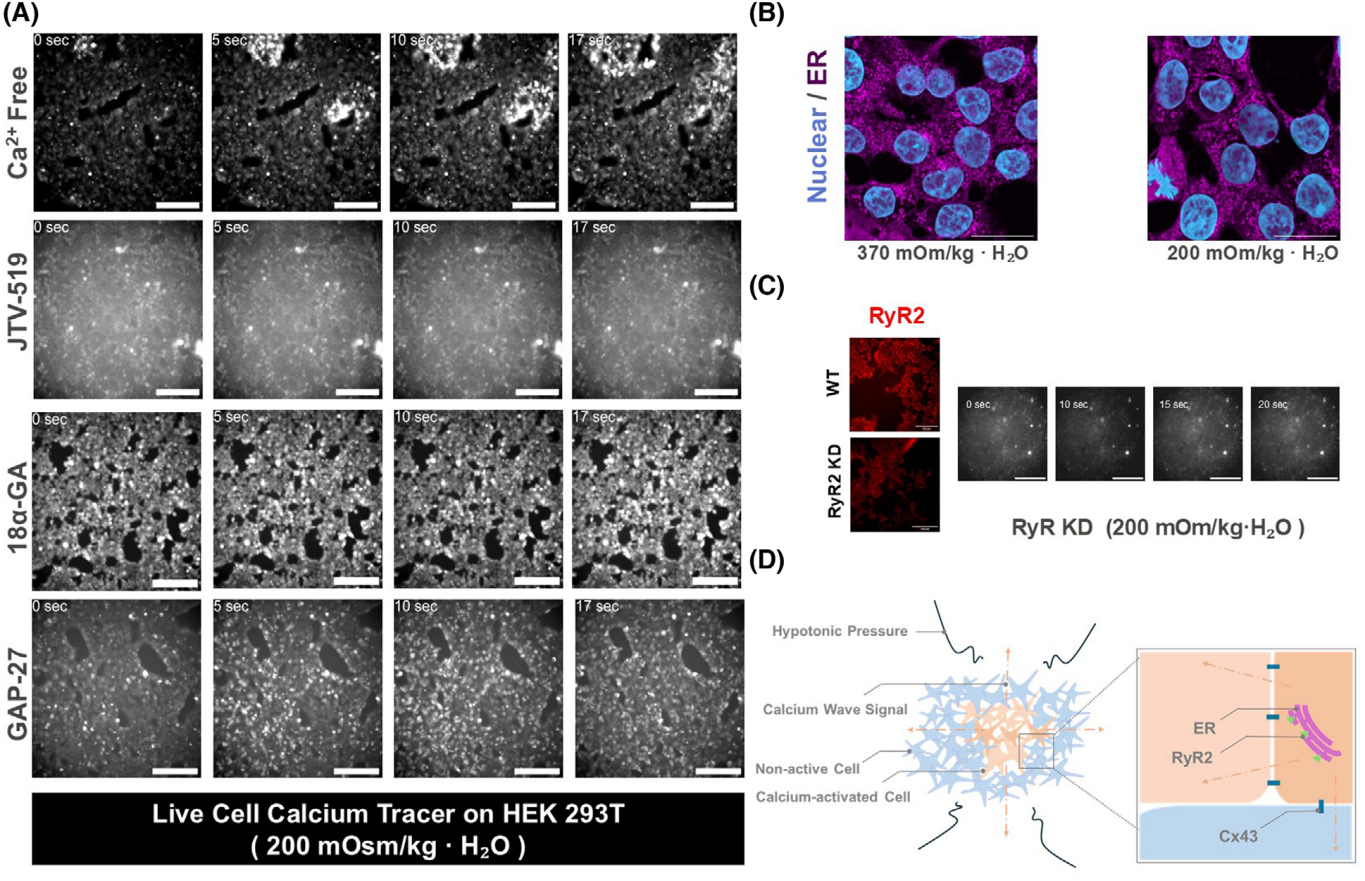
Ryanodine receptor 2 (RyR2)‐mediated intracellular calcium wave activation under hypotonic stimulation in HEK293T cells. (A) The calcium (−) DMEM still induced a calcium wave activation mode. Blocking of RyR2 with JTV‐519 and gap junction with 18α‐glycyrrhetinic acid (18α‐GA) and Gap 27 inhibited wave activation mode in HEK293T cells at ~200 mOsm·kg^−1^·H_2_O. The scale bar is 200 μm. (B) Endoplasmic reticulum (ER) was highly expressed in HEK293T cells. The morphology of the endoplasmic reticulum (magenta) and nucleus (cyan) remained unchanged under a ~200 mOsm·kg^−1^·H_2_O solution compared with the isotonic condition. The scale bar is 20 μm. (C) RyR2 KD HEK293T cells showed that RyR2 (red) signal intensity was reduced compared with wild‐type (WT) cells (left figures). The calcium waves activation mode was not observed in RyR2 KD HEK293T cells at a solution of ~200 mOsm·kg^−1^·H_2_O (right figures). The scale bar is 100 μm on the left figures and 200 μm on the right figs. (D) Under hypotonic pressure, the Ca^2+^ flux did not come from the extracellular solution via channel molecules on the cell membrane, but from the endoplasmic reticulum via ryanodine receptors.

We consider the mechanism of the calcium wave activation mode. Since the cells showed a reliable calcium wave activation mode, we selected the ~200 mOsm·kg^−1^ H_2_O condition in the following experiments. Blocking of membrane channels did not inhibit the calcium wave activation mode (Table 2). Furthermore, we still observed the wave activation mode if calcium‐free medium was used (Fig. 4A, Table 2, Fig. S4). This result suggests that calcium influx originates from the intracellular regions. The ER is one of the calcium storage sites. The ERs were widely distributed in the cytoplasmic region of HEK 293 T cells (Fig. 4B). However, the morphology of the ER under ~200 mOsm·kg^−1^ H_2_O hypotonic solution does not change compared with ~370 mOsm·kg^−1^ H_2_O isotonic solution (Fig. 4B). Ryanodine receptor 2 (RyR2) is a calcium release channel located on the ER membrane. Blocking of RyR2 with JTV‐519 inhibited the wave activation mode (Fig. 4A, Table 2, Fig. S4). We also observed that reducing the expression level of RyR2 by siRNA leads to a decreased rate of calcium wave activation mode (Fig. 4C, Figs S5, S6), reinforcing its critical function. Cell–cell communication is required for the calcium wave activation mode. Gap 27 and 18‐glycyrrhetinic acid, a blocking connexin 43 and a gap junction protein, inhibited the acute calcium wave activation mode (Fig. 4A, Table 2, Fig. S4).

**Table 2:** Inhibitors and other experiments conditions.

Items	Main target channel	Final MW	Incubator time	Calcium wave
D‐MEM (Ca^2+^ free)	NA	10 μm	30 min	⚪
EGTA	Calcium chelator	5 mm	30 min	⚪
Verapamil	L‐Type VGCC	20 μm	30 min	▲
Meclofenamate	L‐Type VGCC	1 μm	30 min	▲
L‐Ascorbic acid	T‐Type VGCC	1 mm	30 min	▲
Di‐4‐ANEPPS	Voltage‐sensitive fluorescent dye	10 μm	30 min	⚪
GsMtx4	SACs	10 μm	1 h	⚪
GsMtx4*	SACs	10 μm	1 h	⚪
Y27632	MLC	10 μm	1 h	⚪
ML‐7	Phosphorylation of MLC	10 μm	30 min	⚪
Blebbistatin	Myosin II	10 μm	30 min	⚪
Cytochalasin D	Actin microfilaments	1 μm	2 min	⚪
cyclosporin A	MTPT	1 μm	10 min	⚪
CGP‐37157	Na^+^‐Ca^2+^ exchanger	20 μm	30 min	⚪
Oligomycin	ATPase	30 μm	10 min	⚪
FCCP	ATPase	20 μm	45 min	⚪
Bongkrekic acid Triammonium salt	ANT	10 μm	10 min	⚪
Ruthenium red	RyR	10 μm	1 h	⚪
Dantrolene	RyR	100 μm	30 min	▲
JTV‐519 (K201)	RyR2	100 μm	1 h	●
S107	RyR2	10 μm	1 h	⚪
U73122	PLC	30 μm	5 min	⚪
Xestospingin C	IP_3_R	100 μm	1 h	⚪
LY294002	PI3K/AKT	0.1 mm	30 min	⚪
Glycyrrhetinic acid	Gap junction etc.	0.1 mm	30 min	▲
18‐Glycyrrhetinic acid	Gap junction etc.	50 μm	3 min	●
Gap27	Cx43	100 μm	3 min	●
Others				
Piezo1 KO HEK293T	NA	NA	NA	⚪
Hypoxic preconditioning	Hypoxia‐related pathways	NA	16 h	⚪

The inhibitors' working concentration and incubation time are shown in the tables.

‘▲’, limit calcium activation; ‘○’, calcium wave or calcium activation; ‘●’, no calcium wave; ANT, adenine nucleotide translocase; Cx43, connexin 43; EGTA, ethylene glycol‐bis(β‐aminoethyl ether)‐N,N,N′,N′‐tetraacetic acid; FCCP, carbonyl cyanide‐p‐trifluoromethoxyphenylhydrazone; HEK293T, human embryonic kidney cells 293 T; KO, knockout; MLC, myosin light chain; MPTP, mitochondrial permeability transition pore; NA, not applicable; PI3K/AKT, phosphoinositide 3‐kinase/protein kinase B pathway; PLC, phospholipase C; RyR, ryanodine receptor; SACs, selective inhibitor of stretch‐activated ion channels; VGCC, voltage‐gated calcium channel. ‘*’ Indicates a different GsMtx4 supplier.

These results suggest that low osmotic pressure propagates [Ca^2+^]i increase signal through gap junctions. Our findings indicate that calcium release from the ER through RyR2 drives calcium wave activation mode in response to acute hypotonic osmolarity and propagates the [Ca^2+^]i increase signal to other cells through gap junctions (Fig. 4D).

## Discussion

As an intracellular second messenger, calcium plays a remarkably diverse role in various biological processes [20] and can be mobilized from both extracellular and intracellular sources [21]. Our results showed that calcium mediated the signal transduction induced by osmotic stress. OSCARS observed here should be related to the protective states for cells [22, 23]. HEK 293 T cells exhibited rapid calcium wave activation mode in response to lower osmotic changes, but not to hypertonic conditions. The silent mode of OSCAR has been reported in other studies [24].

In general, external stimuli activate ion channels on cell membranes, inducing calcium flux from the extracellular matrix. The voltage‐gated calcium channels are the primary regulators of calcium entry [25]. This influx triggers calcium release from the sarcoplasmic reticulum (SR) or ER [26]. A process known as calcium‐induced calcium release (CICR) is critical for muscle contraction [27, 28]. However, in our study, calcium wave activation mode was still observed in the absence of extracellular Ca^2+^. Calcium entry is not essential for calcium wave activation mode. A previous study also reported that calcium oscillations originate from the ER [29]. The ER is the prominent calcium‐storing organelle in the cell. Our results showed that the ER in HEK293T cells was widely distributed, which may explain its sensitivity to osmotic pressure stress. Several kinds of stress induce calcium release from intracellular stores, such as the ER [29]. Osmotic pressure stimulation may be one of the ER stresses.

The function of ryanodine receptor is a key molecule of the calcium release channel in response to mechanical stress, although the role in mechanosensing has not been fully established [30]. Our results indicate that RyR2 is essential to calcium wave activation mode. The Ca^2+^ is also released through IP_3_R in the ER membrane [20]. However, inhibiting IP3R cannot prevent calcium wave activation mode (Table 2). Calcium activation wave mode is effective for signal transduction to neighboring cells [31]. Gap junctions and Cx43 naturally correlate with cell signaling, facilitating cell communication. Our results demonstrate that gap junction families, especially Cx43, play critical roles in propagating calcium activation signaling. Calcium wave activation mode appears to be an exceptional form of calcium elevation and transmission. The velocity of the wave was independent of osmolarity. Compared with our wave activation velocity (12.3 ± 3.9 μm·s^−1^), other cells showed waves that were slower, at 5–8 μm·s^−1^ [32]. It could be due to the different cell types [16, 17, 18, 19]. At 120 mOsm·kg^−1^ H_2_O, the ratio of wave activation mode decreased (Fig. 2B). Under this condition, mechanosensitive channels, such as Piezo1, were activated [29] and increased [Ca]_i_ before ER activation. This may account for our observation that Piezo1 knockout (KO) cells exhibited 100 % calcium wave activation mode at 120 mOsm·kg^−1^ H_2_O (Fig. S7).

Our results demonstrated that calcium serves as a second messenger in response to changes in osmotic pressure in HEK293T cells, confirming that hypotonic conditions trigger rapid calcium release through RyR2 on the ER, which then propagates to neighboring cells via Cx43. RyR2 is a key molecule triggering an osmotic‐related calcium wave. Calcium wave activation mode is the most special form of OSCARS. These findings contribute to the broader understanding of how cells respond to osmotic challenges and may inform future studies of epithelial coordination, tissue‐level homeostasis, or pathological states involving dysregulated calcium signaling.

## Conflict of interest

The authors declare no conflict of interest.

## Author contributions

MM designed the project. GZD and MM constructed all experiments and analyzed the results. GZD wrote the original manuscript, and MM edited it. All authors discussed the results. KN supervised the study.

## Supporting information


**Fig. S1.** Quantitative analysis of cell area stimulated by osmotic pressure.
**Fig. S2.** The fluorescence intensity reflects calcium response modes.
**Fig. S3.** Osmotic pressure‐induced intracellular calcium concentration across cell types.
**Fig. S4.** Effects of inhibitors on calcium wave activation mode.
**Fig. S5.** The knockdown levels of RyR2 were confirmed by RT‐PCR.
**Fig. S6.** The fluorescence images of intracellular calcium concentration in RyR2 knockdown HEK293T cells.
**Fig. S7.** Calcium wave activation rate in Piezo1 KO cells under hypotonic stress.

**Video S1.** Imaging of calcium wave activation mode.

## Data Availability

The datasets supporting the findings of this study have been deposited in the Dryad Digital Repository: https://doi.org/10.5061/dryad.djh9w0wbg. Additional information is available from the corresponding author upon reasonable request.
