# Supplementary material for: Osmotic pressure‐induced calcium response states
Source: FEBS Open Bio. 2025 Aug 29;15(10):1714–22. doi: 10.1002/2211-5463.70094 (PMC12485884; doi:10.1002/2211-5463.70094)
Supplement: Supplementary file 1 — Fig. S1. Quantitative analysis of cell area stimulated by osmotic pressure. Fig. S2. The fluorescence intensity reflects calcium response modes. Fig. S3. Osmotic pressure‐induced intracellular calcium concentration across cell types. Fig. S4. Effects of inhibitors on calcium wave activation mode. Fig. S5. The knockdown levels of RyR2 were confirmed by RT‐PCR. Fig. S6. The fluorescence images of intracellular calcium concentration in RyR2 knockdown HEK293T cells. Fig. S7. Calcium wave activation rate in Piezo1 KO cells under hypotonic stress. [file FEB4-15-1714-s001.pdf]

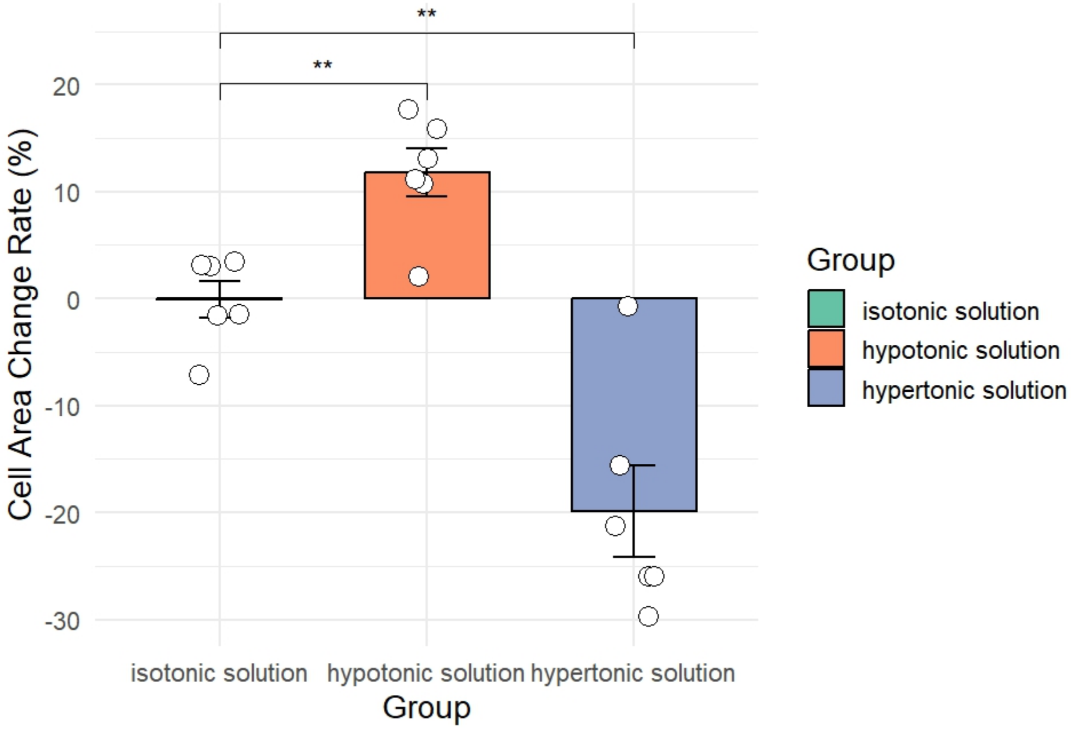

Supplementary Figure 1. Quantitative analysis of cell area stimulated by osmotic pressure

Significant changes in cell area were observed following osmotic change (one-way ANOVA with Tukey's post-hoc test, mean ± SEM, n=6: **p<0.01). Error bars represent SEM.

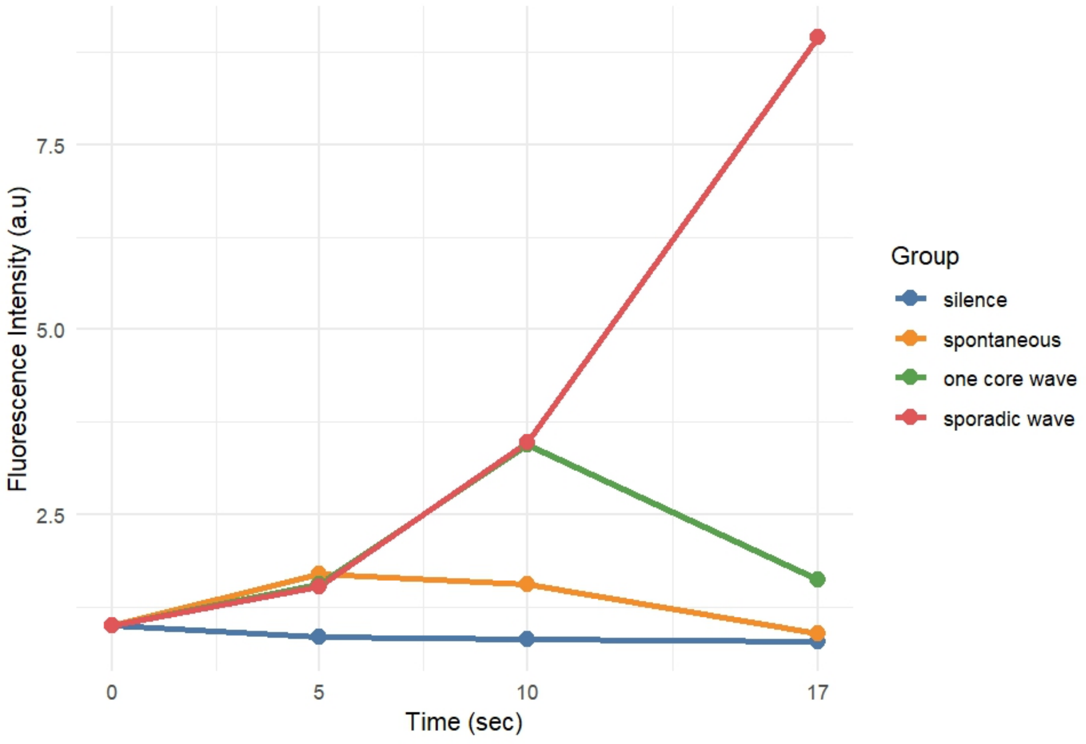

Supplementary Figure 2. The fluorescence intensity reflects calcium response modes

Fluorescence intensity was recorded at multiple time points, showing that the HEK293T cell responds to distinct osmotic pressures in 4 different patterns (for three calcium activation modes).

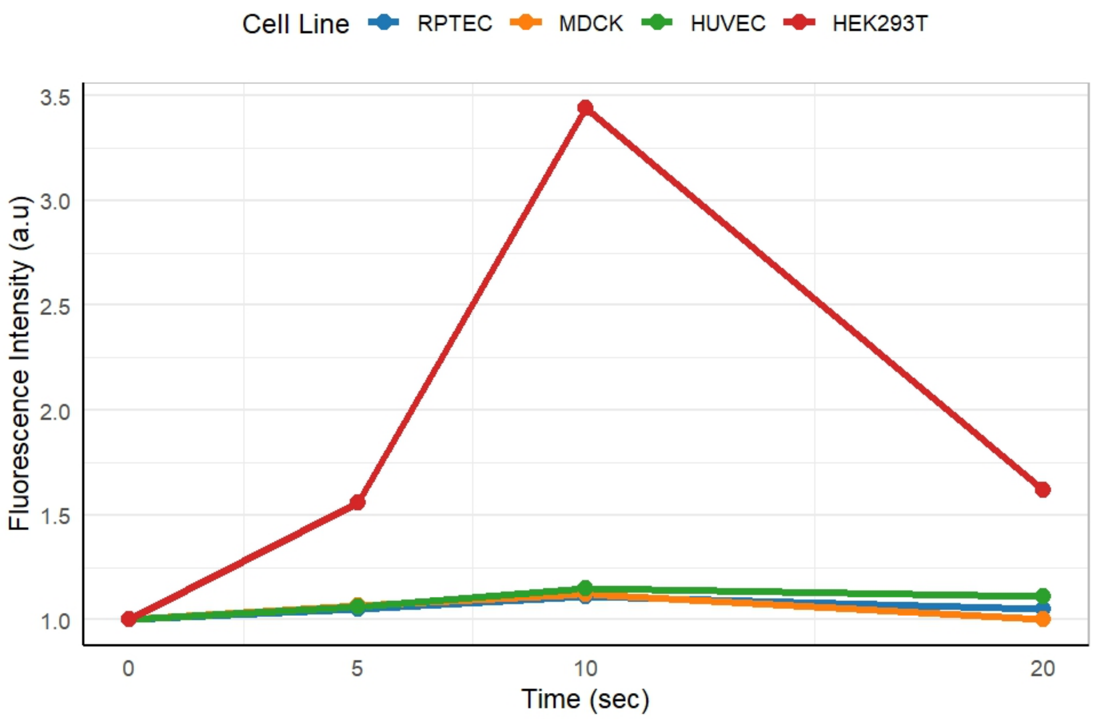

Supplementary Figure 3. Osmotic pressure-induced intracellular calcium concentration across cell types

The fluorescence intensity of intracellular calcium concentration was monitored over time in HEK293T, RPTEC, MDCK, and HUVEC cells, revealing a gradual change in response to osmotic pressure stimulation in different cell types.

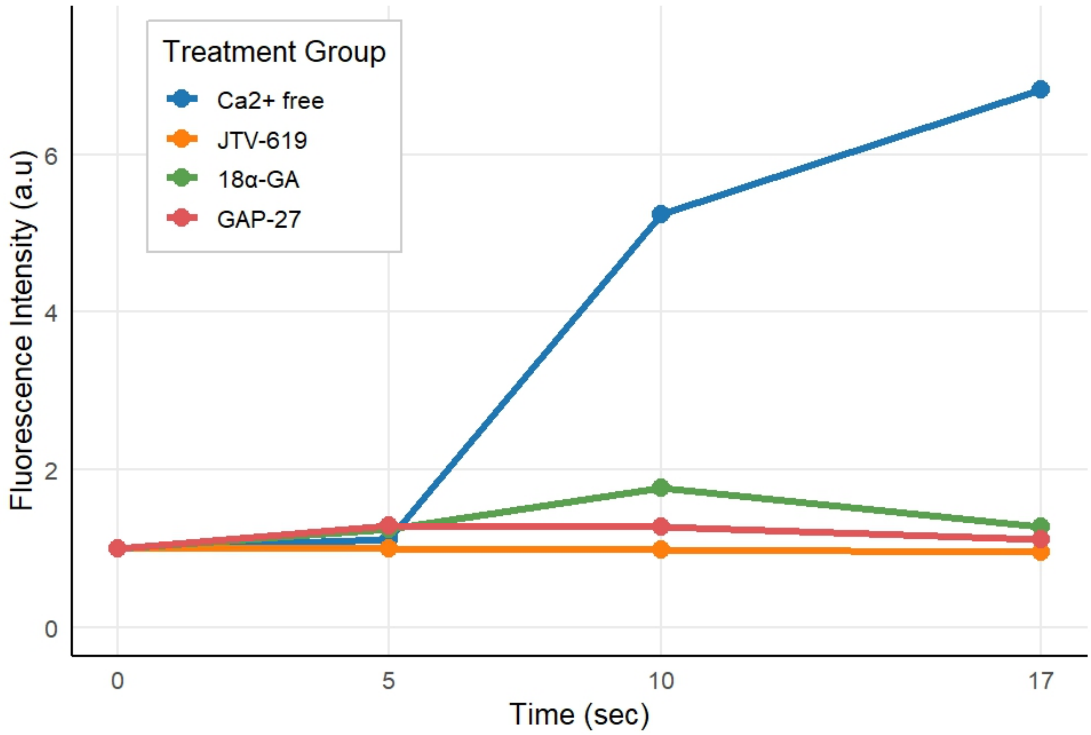

Supplementary Figure 4. Effects of inhibitors on calcium wave activation mode
Fluorescence intensity of intracellular calcium concentration over time in HEK293T cells under various conditions following hypotonic stimulation. A calcium wave activation was observed in calcium-free solution, suggesting intracellular calcium release. Other inhibitors prevented this response, indicating effective suppression of calcium wave activation.

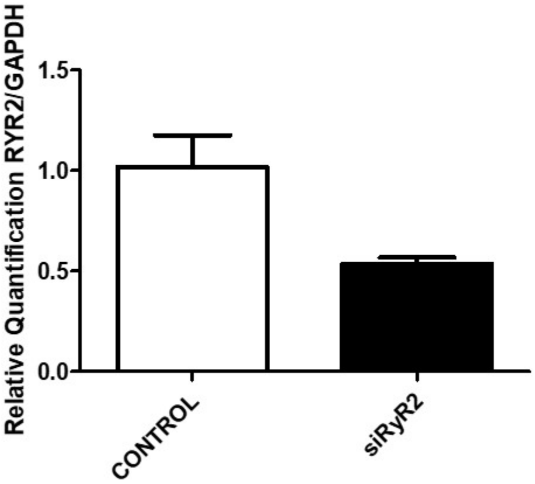

Supplementary Figure 5. The knockdown levels of RyR2 were confirmed by RT-PCR RT-PCR was used to assess the mRNA expression level of the RyR2 gene. RNA was extracted from three biological samples and pooled into one composite sample per group, which was then analyzed in triplicate (technical replicates). The results showed that RyR2 expression was 50 % reduced in the siRyR2-treated group compared to the control group (unpaired two-tailed t-test, mean ± SEM, n = 3). Error bars represent SEM. n represent technical replicates number.

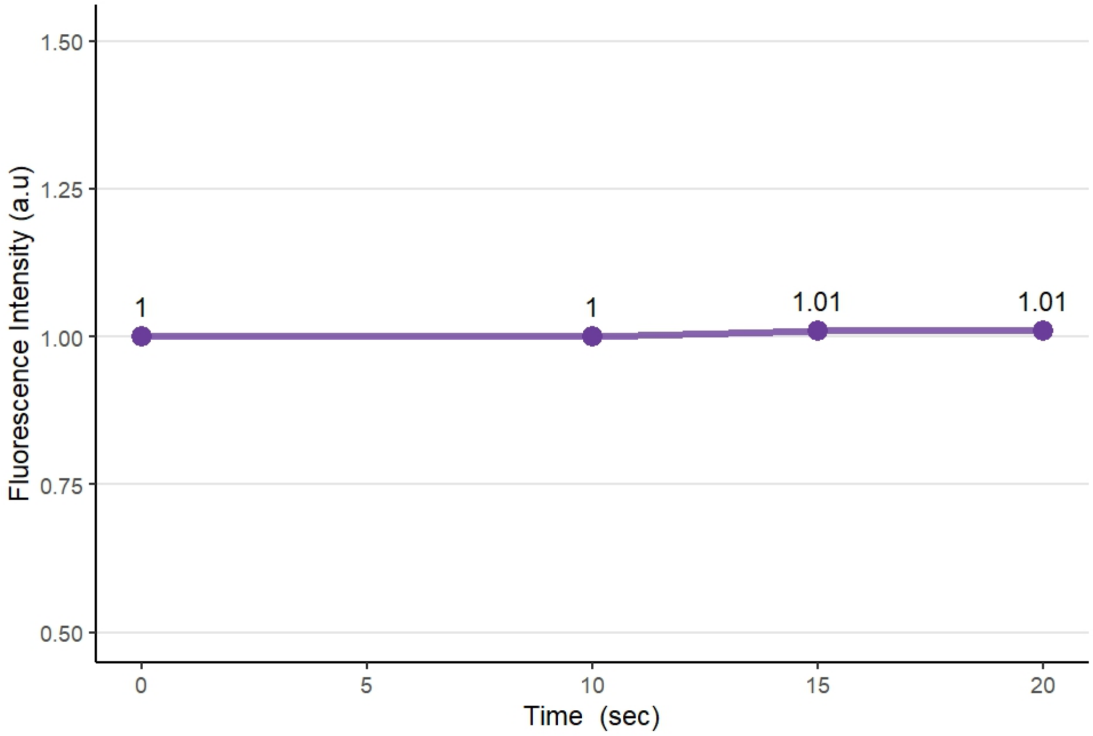

Supplementary Figure 6. The fluorescence images of intracellular calcium concentration in RyR2 knockdown HEK293T cells

Fluorescence intensity of intracellular calcium concentration over time in HEK293T RyR2 knockdown cells under hypotonic pressure. The RyR2 knockdown effectively eliminated calcium wave activation, reinforcing its critical function.

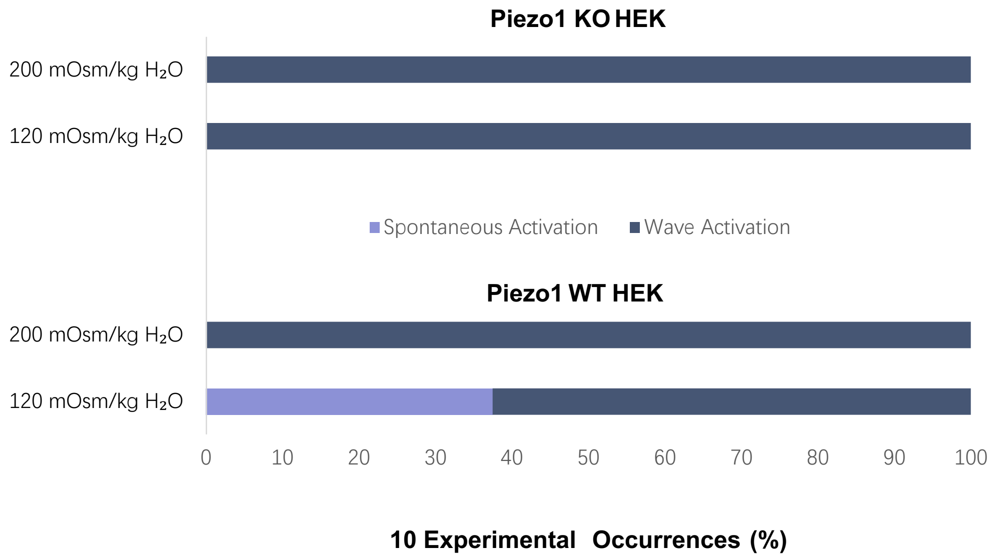

Supplementary Figure 7. Calcium wave activation rate in Piezo1 KO cells under hypotonic stress

Compared with the WT HEK293T cell, the wave activation at 120 mOsm/kg·H_2O solution was decreased, while the KO HEK293T cell showed a 100 % wave activation pattern
